# High dose of epinephrine does not improve survival of children with out-of-hospital cardiac arrest: Results from the French National Cardiac Arrest Registry

**DOI:** 10.3389/fped.2022.978742

**Published:** 2022-10-06

**Authors:** Morgan Recher, Valentine Canon, Marguerite Lockhart, Martin Lafrance, Hervé Hubert, Stéphane Leteurtre

**Affiliations:** ^1^Pediatric Intensive Care Unit, CHU Lille, Lille, France; ^2^ULR 2694 - METRICS: Évaluation des technologies de santé et des pratiques médicales, Univ. Lille, CHU Lille, Lille, France; ^3^French National Out-of-Hospital Cardiac Arrest Registry Research Group - Registre électronique des Arrêts Cardiaques, Lille, France

**Keywords:** out-of-hospital cardiac arrest, cardiopulmonary resuscitation, epinephrine, guidelines, children

## Abstract

**Objectives:**

The pediatric resuscitation guidelines recommend the use of 0. 01 mg kg^−1^ epinephrine during a cardiac arrest; an epinephrine dose higher than that is not recommended. The first aim of this study was to determine the administration rate of high epinephrine dose during pediatric out-of-hospital cardiac arrest. The second aim was to compare the survival status in patients who received high or standard doses of epinephrine.

**Methods:**

This was a multicenter comparative *post-hoc* study conducted between January 2011 and July 2021 based on the French National Cardiac Arrest Registry data. All prepubescent (boys < 12 years old, girls < 10 years old) victims of an out-of-hospital cardiac arrest were included. To compare survival status and control bias, patients who received a high epinephrine dose were matched with those who received a standard epinephrine dose using propensity score matching.

**Results:**

The analysis included 755 patients; 400 (53%) received a high dose and 355 (47%) received a standard dose of epinephrine. The median dose (mg kg^−1^) per bolus was higher in the high-dose group than that in the standard dose group (0.04 vs. 0.01 mg kg^−1^, *P* < 0.001). Before matching, there was no between-group difference in the 30-day survival rate or survival status at hospital discharge. Matching yielded 288 pairs; there was no between-group difference in the 30-day survival rate or survival at hospital discharge (High dose, *n* = 5; standard dose, *n* = 12; Odds ratios: 2.40, 95% confidence interval: 0.85–6.81). Only 2 patients in the standard dose group had a good neurological outcome.

**Conclusion:**

More than 50% of the patients did not receive the recommended epinephrine dose during resuscitation. There was no association between patients receiving a high dose or standard dose of epinephrine with the 30-day survival or survival status at hospital discharge. Collaboration across multiple cardiac arrest registries is needed to study the application of pediatric guidelines.

## Introduction

Pediatric cardiac arrest has a low survival rate and is often associated with a poor neurological outcome (1, 2). The European Resuscitation Council and American Heart Association guidelines for pediatric advanced life support (ALS) during cardiopulmonary resuscitation recommend 0.01 mg kg^−1^ as the standard dose of epinephrine (SDE), with a maximum of 1 mg (3, 4). These guidelines previously recommended 0.01 and 0.01–0.1 mg kg^−1^ for the first and subsequent doses, respectively (5, 6). Using a lower or higher dose of epinephrine (HDE) than 0.01 mg kg^−1^ is not currently recommended, and there is no evidence to support their use in terms of return of spontaneous circulation (ROSC) or prognosis (3, 4).

In a Cochrane review, evidence from studies that compared HDE with SDE in both adult and pediatric populations during in-hospital cardiac arrest (IHCA) and out-of-hospital cardiac arrest (OHCA) was of low quality (10). In the 9 OHCA and IHCA studies on adults, the survival to hospital discharge ranged from 0 to 14% in the HDE group and 0 to 5% in the SDE group. Only 3 pediatric studies were included in the review, with a survival to hospital discharge ranging from 0 to 20% in the HDE group to 0 to 12% in the SDE group (10). These 3 randomized studies found that HDE therapy did not show any benefit over SDE therapy, either during IHCA or in the emergency department after failed prehospital resuscitation (7, 8). Despite the need to follow guidelines during pediatric OHCA, no multicenter study has determined the proportion of patients receiving HDE during OHCA and no study has compared patients receiving HDE and SDE in a prehospital setting.

Using a nationwide French registry of all patients with OHCA, the primary aim of this study was to determine the occurrence rate of HDE during pediatric OHCA. The secondary aim was to compare the survival status in patients in the HDE and SDE groups, before and after adjustment.

## Materials and methods

### Study setting

In France, a medical emergency center is responsible for dispatching emergency professionals [firemen and/or a mobile medical team (MMT)] (9). For OHCA, the prehospital emergency medical system is two-tiered, with fire department ambulances (including a professional first-aid provider) available for prompt intervention and basic life support (BLS) and mobile intensive care units for ALS. Each mobile intensive care unit consists of an MMT that includes a minimum of an ambulance driver, a nurse, and a senior emergency physician.

All participating medical emergency response systems use a specific form to record patient data, time of intervention, type of care, and survival status immediately after medical intervention. The French National OHCA registry (RéAC) forms (www.registreac.org) meet the requirements of French emergency medical organizations. If a patient is alive at hospital admission, a 30 day (D30) or hospital discharge follow-up form must be completed.

### Study design

This multicenter comparative *post-hoc* study analyzed RéAC data collected between January 2011 and July 2021. We determined the occurrence rate of HDE and described patients in the HDE and SDE groups. Then, we compared survival rates in the HDE and SDE groups before and after cohort matching using a propensity score model. The primary endpoint was survival at D30 or hospital discharge. Secondary endpoints were ROSC, 0-day (D0) survival rate, and the neurological outcome at hospital discharge. The neurological outcome was assessed using the cerebral performance category (CPC) score. A favorable neurological outcome was defined as a CPC score of 1–2 (10).

### Eligibility criteria and epinephrine doses

Since 2005, the international guidelines for OHCA use the onset of puberty, the physiological end of childhood, to provide adult care (with 1 mg of epinephrine) to young patients with OHCA (6, 11, 12). The eligibility criteria for this research was prepubescent age, using puberty thresholds to differentiate pediatric and adult care (age: <10 years for girls and <12 years for boys) (13). The following exclusion criteria were applied: dead on MMT arrival; prolonged cardiac arrest (no-flow duration >1 h); spontaneous cardiac activity on MMT arrival; no epinephrine injected; stillbirth; end-of-life care or a do-not-resuscitate directive; and missing mandatory data (e.g., intervention and/or follow-up data).

If the patient's weight was unknown, a theoretical weight was calculated using the weight curves for children (14, 15). We created 2 patient groups according to the dose of epinephrine administered by computing the following:

- recommended per bolus dose based on weight (guidelines, 0.01 mg kg^−1^)
- the actual per bolus dose injected by dividing the total dose by the number of injections and by the patient's weight
- if the real administrated dose was equal to the recommended dose (±20%), the patient was allocated to the SDE group (from 0.008 to 0.012 mg kg^−1^)
- if the administrated dose was more than the recommended dose, the patient was allocated to the HDE group
- if the real administrated dose was less than the recommended dose, the patient was excluded.

#### Data quality control

Several quality control measures are performed on the RéAC database in real time during data input to detect errors, inconsistencies, and out-of-bound values. Offline tests are performed to detect other types of error that require verification from the participating medical emergency response system. Randomly chosen records are assessed by a clinical research associate to identify other inconsistencies or errors that should be included in the automated tests (online or offline).

### Statistical analysis

#### Unadjusted

Normality of distributions was assessed using the Kolmogorov–Smirnov test. Quantitative variables were not normally distributed; hence, they were described as median (interquartile range [QR]). Qualitative variables are shown as frequency. Bivariate analyses were performed using the Pearson chi-squared test for categorical variables and the Mann–Whitney *U* test for continuous variables. Odds ratios (ORs) were calculated for D30 or hospital discharge, ROSC, D0 survival rate, and the neurological outcome at hospital discharge in the 2 groups.

#### Propensity score matching and adjusted data analysis

Propensity scoring was used to reduce the effects of potential confounders on comparisons of the OHCA groups that were treated with HDE or SDE. Propensity scores were estimated using non-parsimonious logistic regression with patient group (HDE or SDE) as the dependent variable and 13 covariates (no flow duration, year of OHCA, age [<2 years, 2–5, 6 years to onset of puberty], etiology [medical, traumatic, drowning, asphyxia, electrocution, overdose], witness-provided BLS, and injection route). Covariates were selected based on their methodological, statistical, and clinical relevance. Patients in the 2 study groups were matched 1:1 based on the propensity score using the greedy nearest neighbor matching algorithm with a caliper width of 0.2 × the standard deviation of the propensity score to create well-balanced groups (16). To evaluate bias reduction, we calculated the absolute standardized differences (ASD) for covariates after propensity score matching. An ASD of <0.1 was considered small (17). ORs for D30 or hospital discharge, ROSC, D0 survival rate, and the neurological outcome at hospital discharge in the adjusted patient groups were compared using Cox logistic regression stratified according to matching, with a 95% confidence interval (CI).

## Results

### Study population

**Figure 1:**
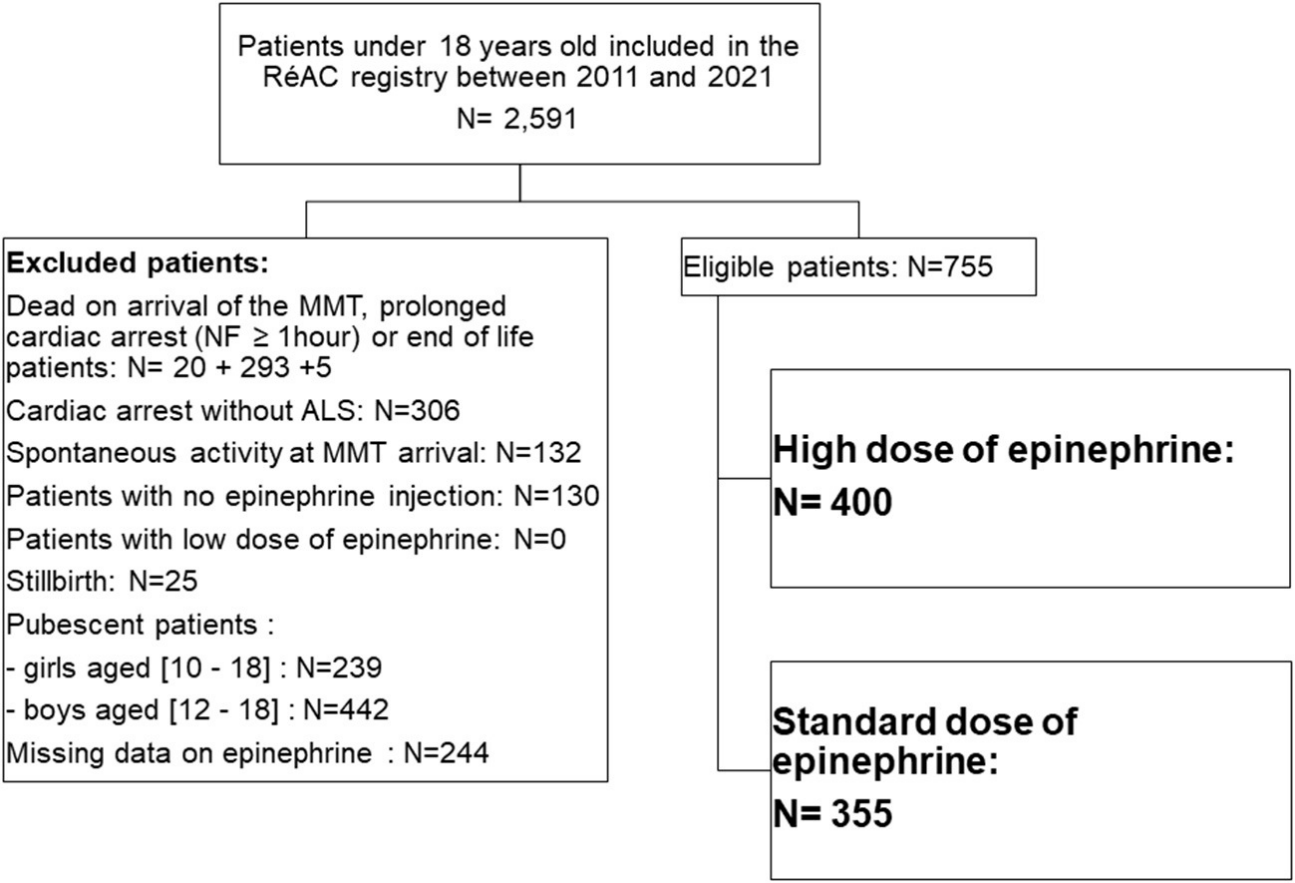
Flow chart of study cohort. ALS, advanced life support; MMT, mobile medical team; NF, no-flow.

We included 755 patients who were treated during the study period (400 in the HDE group and 355 in the SDE group; see Figure 1). No patient in the registry received a dose of epinephrine lower than 0.01 mg kg^−1^. The patient characteristics and medical history are shown in Table 1. Weight for 257 patients was known (HDE group, *n* = 108; SDE group, *n* = 149). The median epinephrine dose per bolus was 0.02 mg kg^−1^ [IQR, 0.01–0.04]. In total, 482 (63.8%) boys were included. The median age was 1 year [IQR, 0–4]. Bystanders were present at OHCA onset in 409 cases (54.2%). Immediate BLS was provided to 247 patients (32.7%). The first rhythm recorded by the MMT was non-shockable in 731 patients (95.6%). The median time from call to administration of epinephrine was 24 min [IQR, 19–31].

**Table 1:** Characteristics of the study population before matching.

	**Entire cohort** ***n =* 755**	**HDE** ***n =* 400**	**SDE** ***n =* 355**	***p***
**Year of CA:** ***n*** **(%)**				<0.001
Before 2015	261 (34.6)	165 (41.3)	96 (27.0)	
2015 and after	494 (65.4)	235 (58.7)	259 (73.0)	
Age (Years): M [Q1; Q3]	1 [0; 4]	1 [0; 5]	1 [0; 3]	0.346
**Age (categories):** ***n*** **(%)**				<0.001
<2	442 (58.5)	232 (58.0)	210 (59.2)	
[2–5]	174 (23.0)	75 (18.8)	99 (27.9)	
[6-puberty]	139 (18.4)	93 (23.3)	46 (13.0)	
Gender: female: *n* (%)	273 (36.2)	138 (34.5)	135 (38.0)	0.325
**CA location:** ***n*** **(%)**				0.188
Home	552 (73.1)	284 (71.0)	268 (75.5)	
Other	203 (26.9)	116 (29.0)	87 (24.5)	
**Medical history**
Cardiac: *n* (%)	54 (7.2)	31 (7.8)	23 (6.5)	0.572
Respiratory: *n* (%)	45 (6.0)	26 (6.5)	19 (5.4)	0.541
Diabetes: *n* (%)	0 (0.0)	0 (0.0)	0 (0.0)	NA
**Etiology of OHCA:** ***n*** **(%)**
Medical	500 (66.2)	261 (65.3)	239 (67.3)	0.590
Traumatic	100 (13.2)	63 (15.8)	37 (10.4)	0.032
Drowning	81 (10.7)	33 (8.3)	48 (13.5)	0.025
Asphyxia	67 (8.9)	38 (9.5)	29 (8.2)	0.608
Electrocution	1 (0.1)	1 (0.3)	0 (0.0)	0.999
Overdose	6 (0.8)	4 (1.0)	2 (0.6)	0.690
**Patients with known weight:** ***n*** **(%)**	257 (34)	108 (27)	149 (42)	
<–3 SD	29 (3.8)	17 (4.3)	12 (3.4)	
Between −3 SD and −2 SD	11 (1.5)	10 (2.5)	11 (3.1)	
Between −2 SD and 2 SD	196 (26)	77 (19.3)	119 (33.5)	
Between 2 SD and 3 SD	5 (0.7)	1 (0.3)	4 (1.1)	
>3 SD	6 (0.8)	3 (0.8)	3 (0.8)	
Patients' weight (kg)	10.0 [6.0; 16.0]	9.5 [5.5; 18.0]	10.0 [7.0; 15.0]	0.385
**Basic life support**
Bystander's presence: *n* (%)	409 (54.2)	228 (57.0)	181 (51.0)	0.108
Immediate BLS: *n* (%)	247 (32.7)	135 (33.8)	112 (31.5)	0.535
Witnesses BLS: *n* (%)	431 (57.1)	208 (52.0)	223 (62.8)	0.003
**Type of witness BLS**				0.525
CC only	236 (54.8)	116 (55.8)	120 (53.8)	
CC + MtM	195 (45.2)	92 (44.2)	103 (46.2)	
1st aid provider BLS: *n* (%)	682 (90.3)	362 (90.5)	320 (90.1)	0.902
AED Shock (before MMT arrival): *n* (%)	24 (4.4)	10 (3.7)	14 (5.0)	0.535
**Advanced life support**
**Initial cardiac rhythm at MMT arrival:** ***n*** **(%)**				0.239
Asystole	695 (92.1)	366 (94.6)	329 (92.9)	
PEA	34 (4.5)	17 (4.4)	17 (4.8)	
VF/pulseless VT	12 (1.6)	4 (1.0)	8 (2.3)	
MMT intubation: *n* (%)	719 (95.2)	381 (95.3)	338 (85.2)	0.999
**Injection route**				0.001
IO	479 (63.4)	232 (58.0)	247 (69.6)	
PIV	276 (36.6)	168 (42.0)	108 (30.4)	
Epinephrine dose (mg): M [Q1; Q3]	1.00 [0.45; 3.00]	2 [0.9; 5.6]	0.6 [0.3; 1.0]	<0.001
Per bolus epinephrine dose (mg/kg)	0.02 [0.01; 0.04]	0.04 [0.02; 0.09]	0.01 [0.01; 0.01]	<0.001
Number of epinephrine injection	6 [4; 10]	6 [3; 10]	6 [4; 10]	0.030
**Care timing**
Time from call (T0) to 1st aid provider arrival: M [Q1; Q3]	9 [6; 13]	10 [6; 13]	9 [5; 12]	0.256
Time from call (T0) to MMT arrival: M [Q1; Q3]	17 [12; 23]	17 [12; 23]	17 [12; 24]	0.567
Time from call (T0) to ROSC or death: M [Q1; Q3]	51 [40; 62]	50 [40; 62]	52 [40; 63]	0.720
Time from call (T0) to adrenaline: M [Q1; Q3]	24 [19; 31]	24 [18; 32]	25 [19; 30]	0.711
No-flow duration: M [Q1; Q3]	6 [0; 12]	7 [1; 12]	5 [0; 12]	0.046
**End of scene**
ROSC	182 (24.1)	95 (23.8)	87 (24.5)	0.865
D0 survival	195 (25.8)	100 (25.0)	95 (26.8)	0.617
D30 survival or HD	24 (3.2)	10 (2.5)	14 (3.9)	0.302
CPC 1–2	4 (0.5)	1 (11.1)	3 (23.1)	0.616

HDE, high dose of epinephrine; SDE, standard dose of epinephrine; CA, cardiac arrest; M [Q1; Q3], median [first and third quartile]; OHCA, out-of-hospital cardiac arrest; BLS, basic life support; CC, chest compressions; MtM, mouth-to-mouth; AED, automatic external defibrillator; MMT, mobile medical team; PEA, pulseless electrical activity; VF, ventricular fibrillation; VT, ventricular tachycardia; IO, intra-osseous; PIV, peripheral intravenous; ROSC, return of spontaneous circulation; D0, day-0; D30, day-30; HD, hospital discharge; CPC, cerebral performance category.

There were no between-group differences in the following variables: median age (1 year), weight, whether BLS was initiated immediately by a witness or first-aid provider using an automated external defibrillator, the initial cardiac rhythm at MMT arrival, intubation rate, time from call to arrival of first-aid, and MMT arrival. The median dose per bolus was higher in the HDE group than in the SDE group (0.04 vs. 0.01 mg kg^−1^, *P* < 0.001). A traumatic etiology of OHCA was more common in the HDE group than in the SDE group (15.8% vs. 10.4%; *P* = 0.032). Drowning and witness-provided BLS were less common in the HDE group than in the SDE group (8.3% vs. 13.5%; *P* = 0.025, 52% vs. 62.8%, *P* = 0.003; respectively). The median no-flow duration was longer in the HDE group than in the SDE group (7 min vs. 5 min, *P* = 0.046). There were no between-differences in time from call to 1^st^ aid provider, to ROSC or death, and to adrenaline.

### Non-adjusted comparison of HDE with SDE

**Figure 2:**
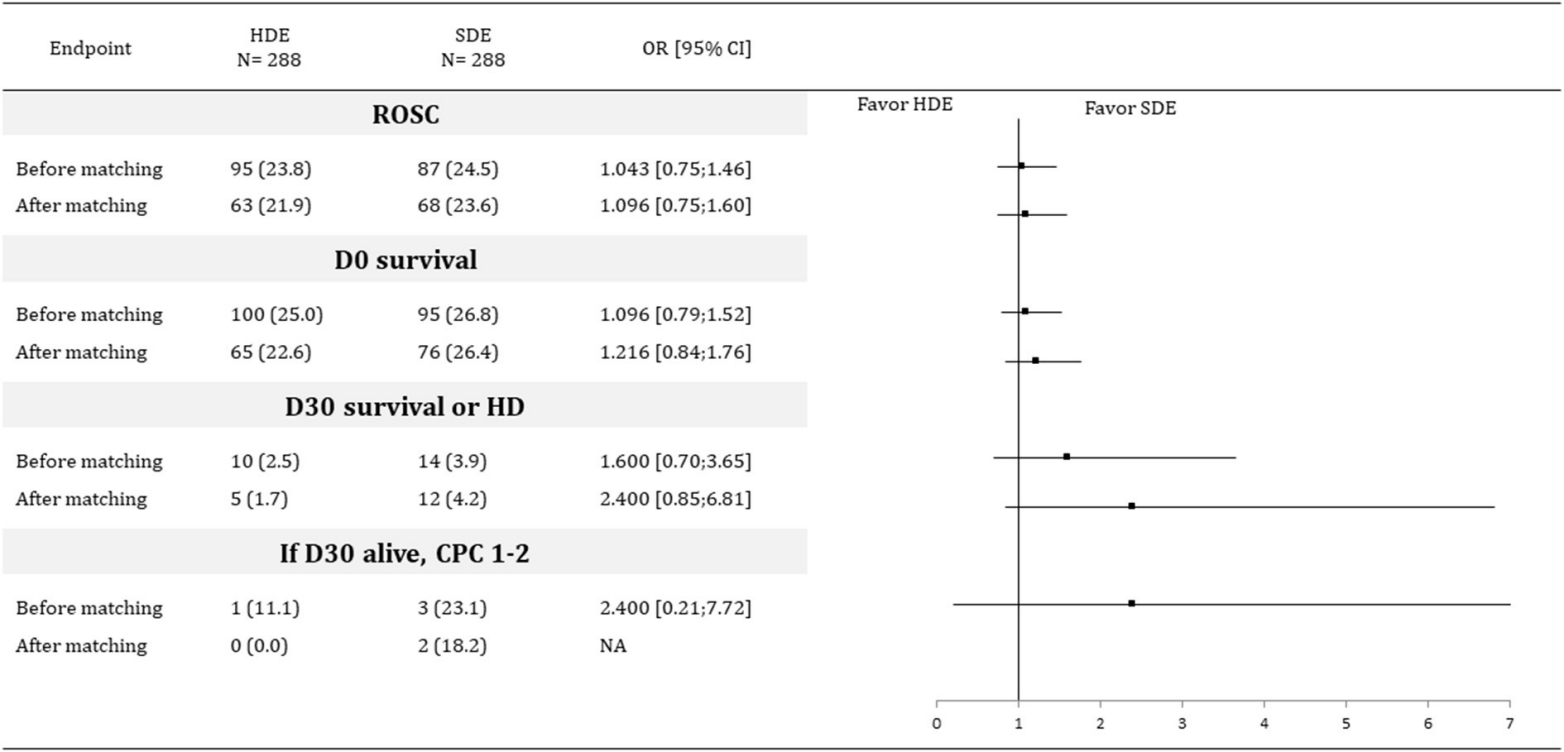
Outcomes before and after propensity score matching. HDE, high dose of epinephrine; SDE, standard dose of epinephrine; OR, odd ratio; CI, confidence interval; CPC, cerebral performance categories; D30, 30 days after ROSC; HD, hospital discharge; OR, odds-ratio; ROSC, return of spontaneous circulation.

After on-scene medical intervention, there were no between-group differences in D30 survival or hospital discharge (2.5% vs. 3.9%; OR: 1.6, 95% CI: 0.70–3.65), in ROSC (23.8% vs. 24.5%; OR: 1.0, 95% CI: 0.75–1.46), and D0 survival (25% vs. 26.8%; OR: 1.1, 95% CI: 0.79–1.52), with 10 and 14 survivors in the HDE and SDE groups, respectively. The neurological outcome was good in 1/14 survivors in the HDE group and 3/10 survivors in the SDE group (Figure 2).

### Adjusted comparison of HDE with SDE

**Figure 3:**
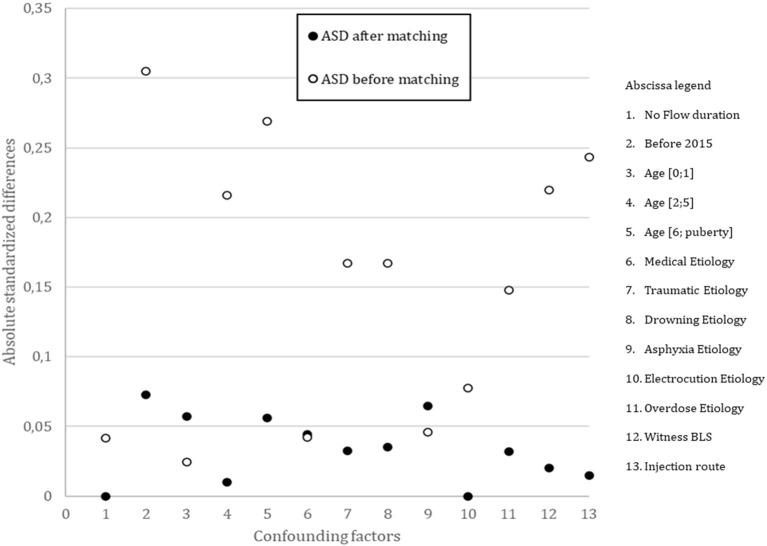
Absolute Standardized Differences (ASD) between groups before and after propensity score matching. BLS, basic life support.

After adjusting for propensity scores (Figure 3), the ASD were <0.1 for each variable. Matching created 288 pairs. There were no between-group differences in D30 survival or hospital discharge (1.7% vs. 4.2%; OR: 2.4, 95% CI: 0.84–6.81), in ROSC (21.9% vs. 23.6%; OR: 1.1, 95% CI: 0.75–1.60), and D0 survival (22.6% vs. 26.4%; OR: 1.2, 95% CI: 0.84–1.76), with 5 and 12 survivors in the HDE and SDE groups, respectively. No neurological outcome was favorable in the 5 survivors in the HDE group, whereas 2/12 survivors in the SDE group had a favorable neurologic outcome (Figure 2).

## Discussion

To the best of our knowledge, this is the first multicenter retrospective study to determine the occurrence rate of HDE during pediatric OHCA and investigate the impact of HDE and SDE therapy on the survival rate in comparable pediatric OHCA populations. No patient in our registry received a low dose of epinephrine and over 50% of patients received a HDE during OHCA. We did not find any between-group differences in survival rate at D30 or hospital discharge, before and after matching. The small number of survivors makes it extremely difficult to draw any conclusions regarding positive prognostic factors for OHCA.

The use of adrenaline has been discussed and challenged for some years. Many studies and reviews of the literature have questioned the effect of adrenaline on survival from OHCA (18, 19). Most observational studies in adult have shown a positive effect of administration of epinephrine on ROSC, but results concerning long-term survival and neurological status are conflicting (18–23). The American heart association advises that it is “reasonable to administer” epinephrine during pediatric cardiac arrest (24). Use of a higher bolus dose than 0.01 mg kg^−1^ every 3–5 min is not recommended because it does not improve survival or the neurological outcome (3, 12). However, the presently recommended epinephrine dose is based on the results of a limited number of pediatric studies (25).

Some retrospective studies have investigated HDE vs. SDE during pediatric IHCA or OHCA. A study by Goetting and Paradis (25) that included 40 IHCA patients suggested that 0.2 mg kg^−1^ of epinephrine achieved a higher ROSC rate and a better long-term outcome than the SDE. Fourteen of 20 patients given HDE had a ROSC; among the 20, 8 survived to hospital discharge. None of the 20 historical controls who had received SDE as rescue therapy had even a transient ROSC. The HDE was 20 times the SDE. Moreover, this study was performed in patients who remained in arrest after at least 2 SDEs of 0.01 mg kg^−1^. However, in a retrospective IHCA study on 51 patients, Carpenter et al. found that HDE (mean dose ± SD, 0.12 ± 0.05 mg kg^−1^) did not improve short- and long-term survival or neurological outcome compared to SDE; 21 patients were treated with HDE and 30 with SDE (26). Moreover, in a retrospective OHCA study in 1995 included 65 children, Dieckmann et al. found that HDE (mean dose ± SD, 0.19 ± 0.06 mg kg^−1^) did not improve short- and long-term survival or neurological outcome compared to SDE. Forty patients received HDE, 13 received SDE, and 12 did not receive epinephrine at all. One of the 40 patients given an HDE and 1 of the 13 given the SDE survived until hospital admission. The high death rate precluded assessment of the epinephrine dose in relation to outcomes (27). These investigations were limited by the small number of patients (40, 51, and 65 patients, respectively). In this study, we included 755 patients, with a majority treated with an HDE, as also observed in Dieckmann and Carpenter's studies. However, the median HDE dose was lower in our study, but the SDE was similar to that in the aforementioned studies. Besides, differences in terms of management of cardiac arrest in children (such as injection of epinephrine through an endotracheal tube) do not allow comparing survival between these 2 studies and our study.

In a 2004 prospective, randomized, double-blind trial, Perondi et al. compared HDE (0.1 mg kg^−1^) and SDE (0.01 mg kg^−1^) as rescue therapy for IHCA in 68 children after failure of an initial SDE. They found, after adjustment in multiple logistic regression analysis, that patients in the HDE group tended to have a low 24 h survival rate (OR: 7.9, 97.5% CI: 0.9–72.5; *P* = 0.08). None of the 34 patients in the HDE group were alive at hospital discharge as compared with the 4 in the SDE group (7), and the main cause of arrest was respiratory. In our study, 10 (2.5%) patients from the HDE group and 14 (3.9%) from the SDE group were alive at D30 or hospital discharge. The main etiology of the cardiac arrest was medical, including respiratory failure. However, we do not know the proportion of the cardiac arrests of respiratory origin. The same initial rhythm of asystole was common in both studies. Perondi et al. has concluded that HDE may be beneficial in a different population of children, such as those who have more prolonged untreated cardiac arrests, those who have undergone cardiac surgery, and those in ventricular fibrillation. Contrary to our study, cardiac arrests occurred in hospitals in their study, where more than 90 percent of patients were monitored and witnessed in the intensive care unit. Most of the patients were receiving mechanical ventilation before the cardiac arrest, and many were already receiving catecholamine infusions.

In a prospective, randomized study including pediatric patients aged from birth to 22 years, Patterson et al. compared the effectiveness in emergency departments of HDE vs. SDE in patients with medical and traumatic OHCA refractory to prehospital resuscitation efforts. One hundred and twenty-seven patients were attended after receiving HDE (0.1 mg kg^−1^ as the initial dose and 0.2 mg kg^−1^ for subsequent doses), and 86 received the SDE (0.01 mg kg^−1^ as the initial dose and 0.02 mg kg^−1^ for subsequent doses). They concluded that HDE did not improve survival to discharge or neurologic outcome, whereas there has been a trend toward increased ROSC rates in HDE medical patients (8). In our study, SDE was defined as 0.008–0.012 mg kg^−1^, lower than that in Patterson's study. However, our conclusions remain similar.

Contrary to our study with 3.1% survival at D30 or hospital discharge, the Young's study, including 599 patients with OHCA, demonstrated an 8.6% long-term survivor rate. Half of the survivors required no epinephrine (28). Engdahl et al. confirmed only a 5% survival rate of OHCA, and Patterson et al. showed a 7.1% survival (8, 29). Previous studies of patients with IHCA have shown somewhat better results with survival rates of 15–54% (30, 31). However, the study by Perondi et al. only showed a 6% survival rate (7). Moreover, only 4 patients in our cohort experienced favorable neurologic recovery. Similar results were found by Patterson et al., with 2/11 survivors with a good neurological outcome and Engdahl et al., with only 3 patients with good neurological outcome (8, 29). The outcome of pediatric cardiac arrest, particularly OHCA, remains unfavorable. The question is how to better identify patients during resuscitation who are unlikely to recover neurologically.

In adult population, there is limited data available regarding the optimal dose and dosing interval of epinephrine during cardiac arrest. Jaeger et al. found, in a retrospective multicenter study using a propensity matching analysis, a negative association with HDE compared to SDE in the D30 survival rate and survival with good neurologic outcomes (32). These results are consistent with experimental and observational studies showing that higher doses of adrenaline result in a poorer survival rate at hospital discharge and neurologic outcome (33, 34). However, adult's etiologies of cardiac arrest differ from those in children, not allowing to compare these populations.

The physiologically beneficial effect of epinephrine is considered to increase diastolic pressure and coronary perfusion pressure through its strong alpha-adrenergic effect, enhances myocardial contractility, stimulates spontaneous contractions, and increases the amplitude and frequency of ventricular fibrillation, so increasing the likelihood of successful defibrillation (3, 21, 35, 36). In contrast, the beta-adrenergic effect can lead to potential harmful effects of epinephrine, which increases myocardial oxygen demand and causes fatal arrhythmia (21, 37). In this way, the HDE could increase the beta-adrenergic effect and the myocardial oxygen demand, resulting in a worse long-term survival and a lower neurologic prognostic. Moreover, through its alpha-1 agonist action, epinephrine administration during resuscitation was shown to reduce cerebral perfusion in animal studies, and contribute to greater neurological injury. Although it initially increases cerebral blood flow, microcirculation is significantly decreased by epinephrine, leading to increased cerebral ischemia (38, 39). Thus, the effect of epinephrine administration on survival and neurologic outcome may be lower than on the chance of ROSC. Given that ROSC is the first step toward survival but that myocardial and brain function have to be preserved, decreasing or spacing adrenaline doses may be questionable.

There is limited research on pediatric OHCA, particularly concerning epinephrine dose. Our study has several strengths. It analyzed data from a nationwide registry covering pediatric OHCA cases. Moreover, our findings are consistent with what previous randomized studies found during IHCA and OHCA refractory to prehospital interventions. Although the epinephrine dose that should be administered during a pediatric cardiac arrest is established, we found that over 50% of pediatric patients with OHCA did not receive the recommended epinephrine dose (3, 4). No changes in guidelines or in practices could explain this. Indeed, the recommended 0.01 mg.kg^−1^ epinephrine dose did not change since 2010, before the onset of our RéAC registry (40), and guidelines are apply throughout France. We did not study if the epinephrine dose was increased at the end of the resuscitation. The duration of resuscitation was not study. However, the time from call to 1st aid provider, and the time from call to ROSC or death were similar in both groups. The length of resuscitation did not appear to be a factor in dose escalation. Further research will be necessary to study the reason not to follow guidelines.

Our study also has several limitations that stem from the nature of the included data. Although the French National Cardiac Arrest Registry is implemented nationwide, not all French emergency medical systems participate. Moreover, due to the geographic diversity of the participating centers, the registry covers a wide range of locations and territories, which permits a comprehensive overview of pediatric OHCA in France. Despite assessment of data quality using randomly selected files and online and offline automatic query systems, the information is gathered in an emergency context and data are reported *a posteriori*. Body weight was known only for 257/755 patients; however, 86.4% had a normal weight with respect to age, and 13.6% were overweight or underweight. We did not perform a sensitivity analysis specifically on these 257 patients. In case of unknown weight, having pediatric specific age-weight formulas or length-weight measuring tapes available for the local out-of-hospital first responders crew could help administer the recommended epinephrine dose. We therefore used the World Health Organization curves to predict the children's weight. We did not perform exact propensity score matching. However, we used a caliper to perform the matching, the use of which has been previously validated (16). Furthermore, we were unable to assess the association of confounding variables, such as comorbidities and premorbid function, with survival. We included patients who received an epinephrine dose of 0.008–0.012 mg kg^−1^ in the recommended dose group. In addition, we did not compare administrated dose in the HDE group to see a difference in survival within this group. Given the marked delay between the first emergency call and epinephrine administration, it is possible that a difference was not detected because epinephrine was administered late to achieve ROSC or affect survival. Pediatric OHCA is a rare event. Despite collecting data, only 288 pediatric cases were included in our matched cohort. As a result, survival outcomes may have been underpowered in the matched analysis. Finally, the matched analysis component might have been underpowered to detect significant differences in survival and neurological outcomes.

In this French national population-based study of pediatric OHCA, more than 50% of patients did not receive the recommended epinephrine dose. There was no association between the HDE and SDE at D30 or hospital discharge survival. Collaboration across multiple cardiac arrest registries is needed to study the application of pediatric guidelines.

## Data availability statement

The original contributions presented in the study are included in the article/supplementary material, further inquiries can be directed to the corresponding author.

## Author contributions

MR and VC conceptualized the study, conducted the initial analyses, drafted the initial manuscript, reviewed, and revised the manuscript. VC and HH performed the statistical analysis and revised the manuscript. MLo, MLa, HH, and SL analyzed and revised the manuscript. All authors have approved the final manuscript as submitted and agreed to be accountable for all aspects of the work.

## Conflict of interest

The authors declare that the research was conducted in the absence of any commercial or financial relationships that could be construed as a potential conflict of interest.

## Publisher's note

All claims expressed in this article are solely those of the authors and do not necessarily represent those of their affiliated organizations, or those of the publisher, the editors and the reviewers. Any product that may be evaluated in this article, or claim that may be made by its manufacturer, is not guaranteed or endorsed by the publisher.
